# An efficiently working brain characterizes higher mental flow that elicits pleasure in Chinese calligraphic handwriting

**DOI:** 10.1093/cercor/bhad047

**Published:** 2023-03-09

**Authors:** Yue Wang, Buxin Han, Min Li, Juan Li, Rui Li

**Affiliations:** CAS Key Laboratory of Mental Health, Institute of Psychology, Beijing, China; Department of Psychology, University of Chinese Academy of Sciences, Beijing, China; CAS Key Laboratory of Mental Health, Institute of Psychology, Beijing, China; Department of Psychology, University of Chinese Academy of Sciences, Beijing, China; Film and TV Centre for Science Popularization, China Science and Technology Museum, Beijing, China; CAS Key Laboratory of Mental Health, Institute of Psychology, Beijing, China; Department of Psychology, University of Chinese Academy of Sciences, Beijing, China; CAS Key Laboratory of Mental Health, Institute of Psychology, Beijing, China; Department of Psychology, University of Chinese Academy of Sciences, Beijing, China

**Keywords:** attention, calligraphy, pleasure, flow, visuomotor

## Abstract

The mental flow that commonly emerges during immersion in artistic activities is beneficial for maintaining mental health. However, there is not that much converging neurobiological evidence about how flow emerges and elicits pleasure in arts. Using an imitation task of Chinese calligraphic handwriting with self-rated subjective flow experience, we investigated the neural interactions supporting flow. Our results show that calligraphic handwriting requires cooperation between widespread multimodal regions that span the visual and sensorimotor areas along the dorsal stream, the top-down attentional control system, and the orbito-affective network. We demonstrate that higher flow is characterized by an efficiently working brain that manifests as less activation particularly in the brain regions within dorsal attention network and functional connectivity between visual and sensorimotor networks in calligraphy. Furthermore, we also propose that pleasure during calligraphy writing arises from efficient cortical activity in the emergence of flow, and the orbito-caudate circuit responsible for feelings of affection. These findings provide new insight into the neuropsychological representations of flow through art, and highlight the potential benefits of artistic activities to boost well-being and prosperity.

## Introduction

Engaging in artistic activities is an increasingly popular way to improve individual well-being. It has been documented that artistic activities can promote cognitive and mental health and have special therapeutic effects on a variety of psychiatric and neurological disorders (Bitonte and De Santo 2014; Yang et al. 2021). For instance, a review of 31 studies suggested overwhelmingly positive cognitive, affective, quality-of-life outcomes benefited from various expressive artistic activities including dance, expressive writing, music, theater arts, and visual arts for older adults (Noice et al. 2014). While most of the early studies have focused on the empirical evidence for the benefits of artistic activities, an increasing number of studies have recently studied the neuropsychological mechanisms underlying the connections between artistic activities and mental health (Blomdahl et al. 2013; Czamanski-Cohen et al. 2019; Lefevre et al. 2020).

The flow experience that commonly emerges during artistic activities is considered foundational for its healing aspects for the mind (Chilton 2013). Flow refers to the mental state of being fully immersed yet effortless in a challenging activity, during which time an individual will hardly tend to be conscious of themselves or their surroundings (Getzels and Csikszentmihalyi 1976; Harris et al. 2017; Van der Linden et al. 2021). Several hypotheses including the transient hypofrontality (Dietrich 2003, 2006), neural efficiency (Grabner et al. 2006; Del Percio et al. 2008), neural proficiency (Bertollo et al. 2016), and synchronization theory (Weber et al. 2009; Huskey et al. 2018a) have been proposed to explain the mental flow. The transient hypofrontality hypothesis suggests that the brief period of suppressed prefrontal activity contributes to flow experience where the explicit analytical system relaxes, whereas implicit command system operates (Dietrich 2003, 2004). It emphasizes that the suppressed activity in the prefrontal cortex, rather than in the whole brain, is associated with flow. However, evidence from functional magnetic resonance imaging (fMRI) studies indicates that the prefrontal cortex exhibits both greater and lower neural activations in different subregions during flow and hypofrontality is not a flow-specific brain state (Ulrich et al. 2014, 2022; Ju and Wallraven 2019). The neural efficiency hypothesis suggests that limited neural resources could be efficiently recruited to consistently experience flow and reduce mental fatigue (Del Percio et al. 2008; Holmes and Wright 2017; Filho et al. 2021), which is characterized by reduced activation in task-related areas (Qiu et al. 2019). However, this assumption has been challenged in moderate to difficult tasks where individuals need to recruit more additional cortical resources to perform at a flow state (Neubauer and Fink 2009; Katahira et al. 2018). The neural proficiency hypothesis and synchronization theory were not confined to use a specific brain region or a single direction of brain activity to explain the neural base of flow. The neural proficiency hypothesis suggests that individuals need to engage in both efficient and effortful control processing to be able to maintain optimal performance. Recently, Filho et al. (2021) suggested that complementary neural mechanisms of transient hypofrontality, neural efficiency, and neural proficiency theoretically contribute to the peak-performance state of flow in sports. The synchronization theory suggests that flow arises from widespread synchronization of cognitive control and reward systems when task difficulty and individual skill are balanced (Weber et al. 2009; Huskey et al. 2018a). Applying network neuroscience approach, the balanced-difficulty condition in video game has been linked to higher average network degree in the frontoparietal control network and a more modular brain network topology, suggesting that the network synchronization corresponds to an energetically efficient brain state for flow (Huskey et al. 2018b, 2022). This energetically optimized system is a reasonable explanation for the distinct characteristics of a sense of high-control and effortlessness during flow. Prominently different from other hypotheses, it particularly emphasizes that the network synchronization should manifest as a pleasurable experience. Huskey et al. (2018a) suggested that the balance between task difficulty and individual ability is associated with high levels of intrinsic reward that corresponds to increased connectivity between cognitive control and reward systems. Apart from these hypotheses, Van der Linden et al. (2021) recently put forward a broad neuroscientific model of flow, suggesting that the behavioral and subjective dimensions of flow are proposed to be linked to the interaction between 3 large-scale networks, namely, the default-mode, central executive and the salience networks. They also proposed that the dopaminergic and noradrenergic systems may be involved in intrinsic motivation and mood states during flow. Previous studies have mostly studied flow in the context of sports, games, and cognitive tasks, but rarely in artistic activities. Neuroimaging studies have demonstrated that artistic engagements commonly require cross-modal interactions of different sensory regions and cooperation of multicomponent cognitive and emotional systems (Herholz and Zatorre 2012; Chen et al. 2017; Thumuluri et al. 2021; Engel et al. 2022). However, it remains less clear how flow is generated from the interplay of these different functional systems during artistic activities.

Most notably, positive emotions and pleasurable experiences that arise from artistic engagement are important contributors to well-being. They are generally linked to dopamine activity in the affective and reward system that includes the orbitofrontal and limbic areas of the brain (Herholz and Zatorre 2012; Wassiliwizky et al. 2017). However, differing from pleasure-only activities, pleasure from art is regarded as a kind of higher-order pleasure that recruits broader neural networks to engage in the attribution of meaning (Christensen 2017). For example, in music and dance (which elicit affective responses), pleasure is triggered by personally meaningful anticipation or prediction process in the sensory areas and association cortices of memory and perception beyond the involvement of the reward system (Zatorre and Salimpoor 2013; Foster Vander Elst et al. 2021). The feelings during flow, such as being fully engaged, able to ignore distractions, perceptions of a merging of action and awareness, a sense of control, and effortlessness, have been suggested to be the basis for the pleasure and meaningfulness experienced at the end of activities (Baker et al. 2015; Bendassolli and Borges-Andrade 2015; Silverman et al. 2016; Van der Linden et al. 2021). Thus, as engaging in artistic activities enables individuals to find a dynamic balance between their personal skills and the task challenges, long-term engagement in artistic activities could result in longer timescales of flow experience, which, in turn, could provide individuals with meaning and purpose, and thus lead to long-term prosperity (Christensen 2017; Stark et al. 2018). However, it is less clear how the neural network interactions that support the flow state stimulate pleasurable emotions during artistic activities.

**Fig. 1:**
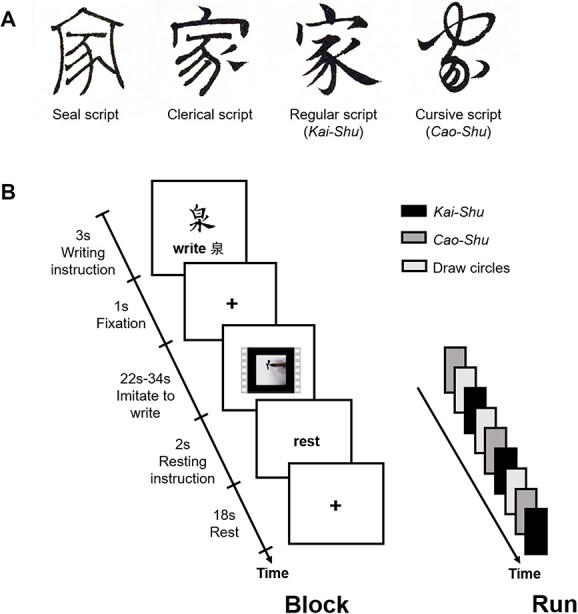
The calligraphy scripts and experiment paradigm. A) Four classic calligraphy styles with the Chinese character “家” (*home*) from the ancient calligrapher Zhao Mengfu’s calligraphy masterpiece as an example. B) Timeline of each writing and drawing task blocks within a functional run, 2 runs in total.

This study uses an imitation task of artistic calligraphy handwriting to investigate how the flow experience emerges from neural network interactions and elicits pleasure. As an ancient expressive art, Chinese calligraphy (also referred to as *Shū-Fǎ*) expresses the abstract beauty of lines and rhythms of Chinese characters through the organizational structure of lines and dots, while also serves the purpose of conveying a person’s thought and emotion. Similar to most artistic activities, it is known as a way to cultivate flow and prosperity in the context of Chinese culture (Chiang 1973). Calligraphy originated from oracle bone inscriptions and has evolved into different calligraphic forms with distinctive rules and esthetic characteristics (Fig. 1A). Traditionally, calligraphy writing needs a soft-tipped writing brush, an ink stick, *Xuan* paper, and an ink-slab as tools, which are known as the “four treasures.” Calligraphy, as a special 3D writing form, requires great manipulation of body parts as well as great intentional concentration. It also requires immediate movement adjustment in accordance with multisensory feedback; for example, the sensory feedback from hands and arms, visual feedback from writing tools, and graphic or operational feedback from writing records on paper (Xu et al. 2013). The dynamic equilibrium between the writer and their brush produces the ability to acquire impetus and produce lively strokes with rhythm and movement (Chiang 1973), and the mentally constructed calligraphy piece can be presented on paper under the guidance of top-down attentional effort. Having clear writing goals and immediate multisensory feedback during calligraphy can help an individual to achieve complete absorption, which screens out irrelevant thoughts and thus contributes to mental flow. Meanwhile, fluent movements and esthetic experiences during calligraphy are thought to benefit emotional expression and elicit pleasure. Calligraphy is thus known as the dance of Chinese characters and the dance of individual spirit.

It is debatable whether flow is an all-or-nothing experience (Michailidis et al. 2018). Some researchers tend to study flow as discrete states (Cairns et al. 2014; Huskey et al. 2018b), whereas others have argued that flow is a continuous spectrum that could characterize the experiential quality (de Manzano et al. 2010; Arzate Cruz and Ramirez Uresti 2017; Frochot et al. 2017). Different from previous studies in which flow was studied by comparing balanced, overload, and bored conditions, degree of flow was measured in calligraphy writing as a continuous variable, and neural correlates of flow were discussed within a dimensional range. Overall, in this study, we would explore the neural mechanism underlying flow in calligraphy and 3 hypotheses were proposed. (1) We first expect that the multi-integrative calligraphy as introduced above will coactivate the multimodal brain systems, including the visual, sensorimotor, top-down attention, and affective networks. (2) Second, if the joint participation of multiple networks represents a busy and focused brain during calligraphy writing, then to meet the immersed and effortless flow experience, an energetically efficient brain would be simultaneously required (Huskey et al. 2018b; Filho et al. 2021). Following the “less is more” operational principle of the brain (Schultz and Cole 2016; Liang et al. 2022), we anticipate that an individual who experiences higher flow will be associated with a less effort/energy demanding (i.e. smaller in amplitude) brain activity pattern. This could be presumably manifested as demonstrating less activation, particularly in the attentional networks, and less interaction strength, particularly between different networks. (3) Third, we hypothesize that the flow experienced during calligraphy will activate pleasure that is beyond the expected direct contribution of the affective regions.

## Materials and methods

### Participants

Nineteen adults were recruited to participate in this study (10 males and 9 females; mean age = 23.47 years, ranging from 20 to 29 years). All participants were right-handed, native Chinese speakers with no formal training in Chinese calligraphy. The participants had normal or corrected-to-normal vision and no history of neurological diseases or psychiatric disorders. The study was performed according to the Declaration of Helsinki and was approved by the Ethics Committee of the Institute of Psychology, Chinese Academy of Sciences (IPCAS). All participants provided written informed consent and received payment for their participation prior to the experiment.

### Experimental stimuli

The stimuli included 16 silent video clips, in which a calligraphy expert used a soft-tipped brush to reproduce calligraphic characters from ancient calligraphy masterpieces, at a slow speed, on 6 × 6 cm red grids on white Chinese art paper (*Xuan* paper). The videos were recorded with a digital camera from a first-person perspective. Using Adobe Premiere Pro 2020, all video clips were edited into squares with equal space around them to highlight the process of the brush moving and minimize the calligraphy expert’s body movement to reduce interference. This process produced 16 video clips that ranged from 22 to 34 s (mean duration = 26 s). Each clip recorded the complete writing process for one Chinese character. A previous study has suggested that even in the context of comparably short activation blocks, flow can be reliably experienced and associated with changes in neural activation (Ulrich et al. 2015).

This study used 2 typographical calligraphic styles: *Kai-Shu* and *Cao-Shu*. *Kai* means “model,” and is a style characterized by squareness and precision. It is inflexible in regularity of design, moderate in difficultness, easy to learn, and is one of the primary styles of learning calligraphy. *Cao* means “grass,” and signifies a “rough” or “care-free” style. It is flexible, uncurbed, and is usually practiced after acquiring basic calligraphy skills (Chiang 1973). When learning calligraphy, *Kai-Shu* is usually practiced first followed by other styles in order of difficulty and preference (Chiang 1973). For the participants who had no long-term experience of calligraphy practice, *Kai-Shu* items were easier to identify and follow, thus achieving a higher level of skill-challenge balance, whereas *Cao-Shu* could result in lower-level balance. Therefore, these 2 typographical calligraphic styles used in this study, on the one hand, could make the experimental materials more representative and reduce selection bias when study flow experience in calligraphy activities as a whole; on the other hand, different levels of skill-challenge balance resulted from the 2 styles could further contribute to investigate the neural mechanism of flow experience.

Eight characters in the *Kai-Shu* style were selected from the ancient calligrapher Ouyang Xun’s calligraphy masterpiece, *Jiuchenggong Liquanming*, which has been praised as “the best *Kai-Shu* in the world.” Another 8 characters in the *Cao-Shu* style were chosen from Wang Xizhi’s masterpiece, *Shiqi Tie*, whose graceful and fluent *Cao-Shu* has dominated the minds of the Chinese for thousands of years. The forms of the selected 16 ancient Chinese characters were the same as those used in today’s simplified Chinese writing system. Moreover, the frequencies of the characters were counted according to database developed by Cai and Byrsbaert (2010), which is based on a corpus of film and television subtitles and exceeds the median frequency of Chinese characters so that unfamiliarity can be avoided. The character lengths (*t* = −0.92, *P* = 0.39), frequencies (*t* = 0.28, *P* = 0.79), and durations of the videos (*t* = −1.51, *P* = 0.18) were balanced between the 2 calligraphic styles (Table S1).

To control for general activation elicited by motor and visual processing, the participants were also told to watch snow-like videos and simultaneously draw circles with their right hand. Circle drawing task has been widely used as a control task for studies of writing in fMRI environment (Rapp and Lipka 2011; Villiger et al. 2013; Yang et al. 2019).

### Experimental paradigm

We used an imagined calligraphy writing experimental paradigm in the form of a typical fMRI block design, in which the participants were instructed to watch the calligraphy writing video clips and imitated writing the visually presented calligraphic character in each block by slightly moving their right hand (Fig. 1B). This imaginative handwriting is very close to the first and foremost “reading” step in learning of model calligraphic works, in which a learner ponders the strokes and structure of characters to be written in a manner of air-writing using their hand or brush without inks. The imagery paradigm has been used in previous studies (Calvo-Merino et al. 2005; Harrington et al. 2007; Wolf et al. 2015), including the study in which imagined table tennis playing was used to explore the relationship between brain asymmetry and flow experience (Wolf et al. 2015). Besides, there is also evidence that motor imagery, observation, and execution share the same neural basis (Caspers et al. 2010; Hardwick et al. 2018). Based on these considerations, we assume imagined calligraphy writing to be an appropriate paradigm with which to test our hypotheses.

The experiment consisted of 6 blocks of imaginary calligraphy writing (3 blocks each of *Kai-Shu* and *Cao-Shu*) and 3 blocks of drawing circles in a pseudorandom order in both runs. The task lasted for 15 min and 10 s. As shown in Fig. 1B, each block began with a 3 s visual presentation of a task that was composed of a picture that displayed either a complete calligraphic character with the instruction to “write X” below, or a snow-like picture with the instruction to “draw circles” below, followed by a 1 s fixation. The instructions encouraged the participants to encode the overall calligraphic character to be written next. Then, the video clips were played. During this time, the participants were either asked to imitate the “writing” and to imagine and replicate the behavior in the videos as though they themselves were manipulating the brush to write the calligraphy, or they were instructed to draw circles while watching snow-like videos. The videos were presented on a computer screen situated outside of an MRI scanner, which the participants viewed via a mirror (12 × 9 cm) that was located inside of the scanner. All blocks ended with an 18 s rest. In the preparation phase before scanning, the participants underwent 6 practice blocks outside the MRI scanner (2 blocks of *Kai-Shu* and *Cao-Shu,* respectively, and 2 blocks of drawing circles), and all procedures were the same as those in the formal experiment.

After the scanning, the participants were immediately instructed to complete 9 statements on a Likert scale; the responses ranged from 1 (“I do not agree at all”) to 7 (“I completely agree”). These items were used to assess the participants’ subjective experiences of flow during the *Kai-Shu* and *Cao-Shu* writing tasks, respectively, in terms of their enjoyment, perceived balance between their skills and the task demands, concentration, clear goals, instant feedback, feeling of control, subjective sense of time, reduction in self-consciousness, and autotelic experience. The 9 items referenced to 9 characteristics of flow and items that have been used in published research (Ullén et al. 2012; Ulrich et al. 2014), and the wording was modified to suit the current study’s focus on the calligraphy writing task. The mean of the 9 items was used as the score of overall flow. The items were: (1) “I enjoyed the calligraphy writing”; (2) “I felt that my ability to complete the calligraphy writing task completely matched the task’s difficulty”; (3) “I felt like I could completely concentrate during the calligraphy writing task”; (4) “I had a clear picture of what I needed to do during the calligraphy writing task”; “(5) I am conscious of how well I have performed in the calligraphy writing tasks”; (6) “I had a sense of complete control during the calligraphy writing task”; (7) “I felt like time passed quickly during the calligraphy writing task”; (8) “During the calligraphy writing task, all thoughts on task-irrelevant issues that personally concerned me were extinguished”; and (9) “I completed the calligraphy writing task spontaneously and automatically without having to think.” The internal consistency reliability of this scale was assessed (Hayes and Coutts 2020), and McDonald’s omega coefficients were considered acceptable (*Kai-Shu*: *ω* = 0.85; *Cao-Shu*: *ω* = 0.81). The participants’ pleasure was determined by asking: “To what extent did the calligraphy writing please you?” The participants gave their responses on a scale ranging from 1 (“not at all”) to 7 (“as much as possible”) based on their subjective feelings about the *Kai-Shu* and *Cao-Shu* tasks, respectively.

### fMRI acquisition

A 3-Tesla GE scanner (GE Discovery MR750, GE Healthcare, Milwaukee, WI, USA) at the IPCAS MRI Research Center was used in the fMRI data acquisition. Functional images were acquired using a blood-oxygen-level-dependent (BOLD)-sensitive T2^*^-weighted, gradient-echo planar imaging sequence with the following parameters: time repetition (TR) = 2,000 ms, time echo = 30 ms, flip angle = 90°, field of view = 224 × 224 mm, slice thickness = 3.5 mm, in-plane resolution = 3.5 × 3.5 mm, matrix = 64 × 64, and 37 axial slices. High spatial resolution anatomical images were acquired using a T1-weighted, spoiled gradient-recalled echo sequence with the following parameters: TR = 2,000 ms, slice thickness = 1.0 mm, in-plane resolution = 1 × 1 mm, matrix = 256 × 256, and flip angle = 12°.

### fMRI data processing and analysis

#### Processing

The fMRI data were preprocessed and analyzed using SPM12 (Wellcome Trust Center for Neuroimaging, London, UK; http://www.fil.ion.ucl.ac.uk) and the CONN toolbox (version 20b; Whitfield-Gabrieli and Nieto-Castanon 2012). The preprocessing was performed following the CONN’s default preprocessing pipeline for volume-based analyses. The pipeline includes functional realignment and unwarping; slice-timing correction; outlier identification by means of the artifact detection tool (ART; note that acquisitions with framewise displacement above 0.9 mm or global signal changes above 5 SD are flagged as potential outliers); direct segmentation of gray matter, white matter, and cerebrospinal fluid, normalized to the standard Montreal Neurological Institute (MNI) space (3 mm^3^); and functional smoothing with a Gaussian kernel of 8 mm full-width at half-maximum. The CONN’s anatomical component-based noise correction procedure (aCompCor) was then implemented to further remove potential confounding effects. This included 5 principal noise components from the cerebral white matter and cerebrospinal areas, respectively; an estimated 12 subject-motion parameters (3 translations, 3 rotations, and associated first-order derivatives); scrubbing parameters of outlier scans, as identified by the ART; and the linear BOLD signal trend within the session. The constant task effects were additionally removed in the aCompCor procedure before entry into the generalized psychophysiological interaction (gPPI) analyses. In a separate step after the aCompCor regression, a temporal band-pass filter was also applied to remove temporal frequencies below 0.008 Hz.

#### Activation analyses

In the first-level individual analysis, activation maps that contrasted the calligraphy writing task with the circle drawing task were generated for each participant using a general linear modeling (GLM). For each participant, a GLM was set up by specifying the onsets and durations of the task blocks for the calligraphy writing and circle drawing as separate regressors. Resulting boxcar functions for each block were convolved with the canonical hemodynamic response function. The second-level group analysis of the brain activation was subsequently performed using a random-effects model and a one-sample *t*-test. The voxel-wise threshold for statistically significant activation was set at a false discovery rate (FDR) and was corrected at *P* < 0.05 for multiple comparisons, with a minimum cluster extent of 20 contiguous voxels. In a similar vein, the contrast between the 2 calligraphic styles (*Cao-Shu* vs. *Kai-Shu*) was also estimated. As their inherent challenges are conducive to investigate flow experience on different levels of challenge-skill balance, after the overall analysis of calligraphy writing tasks, we run contrasts on the 2 styles to see if a higher flow (*Kai-Shu*) is with a less effort/energy demanding (i.e. smaller in amplitude) brain activity pattern.

#### gPPI analyses

To show how the activated regions cooperated during the calligraphy writing task, a generalized gPPI model was conducted to compare the functional connectivity between the calligraphy writing and circle drawing tasks. To avoid selection bias of regions of interest (ROIs), an independent set of ROIs selected from CONN’s network atlas with ROIs characterizing classical networks, closely matching the distribution of main activations in the present study was used. Briefly, 11 ROIs that are from the visual (4 ROIs, including bilateral lateral visual areas (LVA), medial, and occipital visual areas (MVA, OVA)), sensorimotor (3 ROIs, including bilateral and superior sensorimotor areas (SMA)), and dorsal attention (4 ROIs, including bilateral frontal eye fields (FEF) and intraparietal sulcus (IPS)) networks were selected. The BOLD timeseries of each ROI (target) were predicted using the mean timeseries of other ROIs (sources), the handwriting condition regressors as the psychological factors, and the interaction terms of the handwriting conditions and source ROIs timeseries as the psychological modulation factor. The modulatory factor represented a directional effect of the source ROIs on the target ROI; that is, the directed functional connectivity (or effective connectivity) between them. Then, the modulation terms were subjected to the second-level group analysis to evaluate the connectivity difference between the calligraphy writing and circle drawing conditions using paired *t*-tests, thresholded at the connection-level of *P* < 0.05 and FDR-corrected. Consistent with activation analyses, we also assessed the ROI-to-ROI functional connectivity difference between the 2 calligraphic styles (*Cao-Shu* vs. *Kai-Shu*).

#### Multivariate pattern analysis

Considering the above functional connectivity analyses was limited to interactions of activated brain regions, an exploratory, data-driven, whole-brain voxel-wise principal component analysis (PCA)-based multivariate pattern analysis (MVPA) method (Whitfield-Gabrieli and Nieto-Castanon 2012) was used as a complement. MVPA first maps all the multivariate connectivity patterns between all voxels across all subjects and tasks, and then performs a PCA to create a low-dimensional multivariate representation that characterizes the connectivity pattern for each voxel (Whitfield-Gabrieli and Nieto-Castanon 2012; Thompson et al. 2016; Brugman and Burgers 2021). The number of PCA components was set as 2 because it is recommended to use either 1:5 or 1:10 ratios for the number of MVPA components to the number of subjects (Thompson et al. 2016). The results were thresholded at the voxel level of *P* < 0.001 and cluster size > 10, uncorrected. A resultant cluster meant that there was a significant difference in the functional connectivity between this cluster and other areas between the task conditions (i.e. calligraphy writing vs. circle drawing, or *Kai-Shu* vs. *Cao-Shu*). Thereafter, if additional ROIs or networks within our hypotheses were found from the data-driven MVPA, post hoc analyses were followed to further clarify the role of the connectivity of these additional areas.

### Association with self-reported data

We conducted Shapiro–Wilk tests to assess the data normality (i.e. experienced flow and pleasure), then chose nonparametric methods (i.e. Wilcoxon matched-pairs signed-rank test and Spearman correlation analyses) for skewed distributed variables, and parametric methods (i.e. paired samples *t*-tests and Pearson correlation analysis) for normally distributed variables. The results showed that the values for pleasure among the *Kai-Shu* items had a skewed distribution (*P* = 0.039); those in the holistic calligraphy writing task and the *Cao-Shu* items were normally distributed (all *P* > 0.05). The values for flow in the calligraphy writing task and the 2 calligraphic styles were normally distributed (all *P* > 0.05). Therefore, parametric paired-samples *t*-tests were used to compare the differences in the participants’ subjective flow levels between the 2 calligraphic styles. Nonparametric Wilcoxon matched-pairs signed-rank test was utilized to test whether the degrees of pleasure differed between the 2 calligraphic styles. Moreover, Spearman rank correlation analyses were performed to explore the relationship between pleasure in the *Kai-Shu* items and the values for brain activity. Pearson correlation analyses were conducted to reveal the relationship between the rest of the self-reported data and the values for brain activity.

Independent-samples *t*-tests and Spearman correlation analyses were conducted to eliminate the effects of demographic variables on the results. The analysis revealed no significant gender differences for flow level (*t* = −0.69, *P* = 0.50) and experienced pleasure (*t* = −1.34, *P* = 0.20). Years of education, age, and mean head motion had no correlation with the flow level and experienced pleasure (all *P* > 0.13). Therefore, the potential influence of these factors could be excluded.

To test Hypotheses (2) and (3), we conducted a series of correlation analyses between brain activity values and self-reported data using SPSS 25.0 (IBM Corp. in Armonk, NY, USA). Accordingly, we extracted the activation and connectivity values of brain regions involved in calligraphy to establish the relationships with subjective flow or pleasure. Furthermore, we used a probability-based data-driven Gaussian Bayesian network (GBN) approach (Shachter and Kenley 1989; Li et al. 2020) to causally model the relationship between brain activity, flow, and pleasure. The GBN estimates the causal relationships by searching the conditional dependencies or independencies between variables in a global-learning manner. We used the L1-regularization paths algorithm (Schmidt et al. 2007) and maximum likelihood estimate implemented in the Bayes Net Toolbox for MATLAB (Murphy 2001) to learn the structure and parameters of the GBN model. The Bayesian information criterion (BIC; Schwarz 1978) was used to identify the optimal model by selecting the one optimizing the BIC score among the space of GBN graphs.

**Fig. 2:**
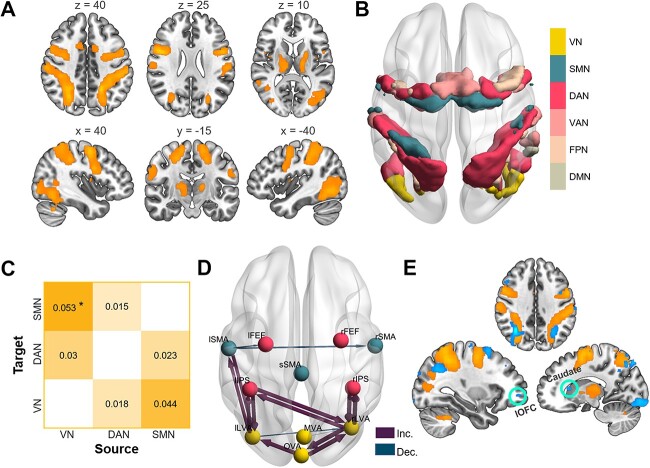
Activation and connectivity in calligraphic handwritings. A) Contrast images with an FDR-corrected voxel *P* < 0.05 display significantly enhanced activation in handwritings compared with circle drawings. B) Spatial distribution of activated cortical voxels in Yeo’s 7-networks. Note that no voxel was found in the Yeo’s limbic network, and voxels shown in the ventral attention, frontoparietal, and default networks were closely connected against edges of core clusters (the IPS and FEF) of the dorsal attention network. C) A schematic depiction of mean change in between-network functional connectivity in handwritings compared with drawing circles (^*^P < 0.05, FDR-corrected). Each cell refers to a directional gPPI connection. D) Change in functional interactions between 11 ROIs in calligraphic handwritings compared with drawing circles. Connections significant at FDR-corrected *P* < 0.05 were shown. E) Whole-brain voxel-wise multivariate correlation exploration using the MVPA (uncorrected voxel *P* < 0.001) displays additional involvement of the OFC and caudate from OAN in handwritings. For visual comparison, the MVPA pattern (cold color) was overlapped on the activation contrast (warm color). Inc. = increased gPPI connectivity, Dec. = decreased gPPI connectivity, DAN = dorsal attention network, VAN = ventral attention network, FPN = frontoparietal network, DMN = default-mode network, sSMA = superior SMA. Volume and surface visualizations were made, respectively, by MRIcroGL and BrainNet Viewer.

## Results

### Coactivation and cooperation of multi-networks during calligraphy writing

The contrast testing for significantly greater activation during calligraphy writing compared with control condition yielded widespread brain regions, including the bilateral occipitotemporal cortex, precentral/postcentral gyrus, IPS, FEF, and putamen/thalamus (*P* < 0.05, FDR corrected; Fig. 2A and Table S2). As anticipated, the calligraphy writing task recruited a widely distributed set of brain regions that mainly spanned the visual (VN), sensorimotor (SMN), and dorsal attention (DAN) networks as visually confirmed by Yeo’s brain network parcellations (Fig. 2B; Yeo et al. 2011).

By averaging the timeseries of the ROIs within each network to perform a network-level gPPI analysis first, we found that compared with the circle drawing task, the calligraphy writing task resulted in increased functional connectivity from the VN to SMN (*P* = 0.04, *t* (18) = 3.05, FDR corrected; Fig. 2C). In the subsequent ROI-level gPPI exploration, a total of 11 and 4 connections showed enhanced and weakened connectivity, respectively, during the calligraphy writing task (*P* < 0.05, FDR corrected; Fig. 2D). Specifically, 8 of the 11 increased connections were between-network interactions of the visual areas with the sensorimotor and dorsal attention networks; that is, the bidirectional interactions between the left LVA with the left SMA and the left IPS (DAN), and between the right LVA with the bilateral IPS (DAN). The remaining 3 increased connections were within-VN interactions, i.e. the connectivity between the occipital and bilateral lateral occipital ROIs. Regarding the decreased connectivity, except for the connection from the left SMA to the left FEF of the DAN, the other 3 within-network connections were in the VN and SMN, including the connections from the left lateral and medial visual area to the right LVA, and between the left and right SMA.

**Fig. 3:**
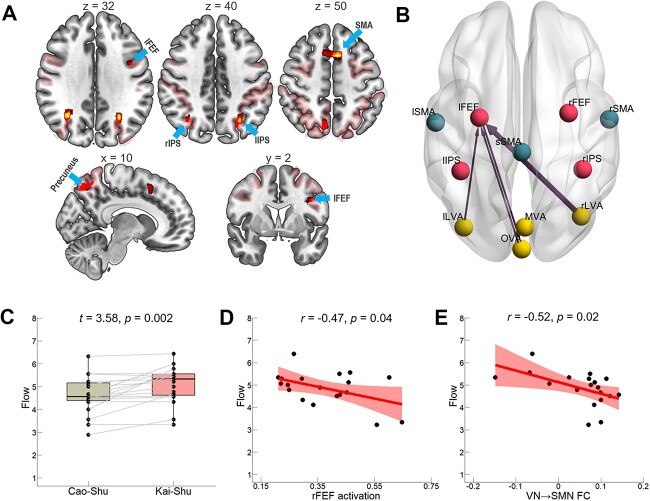
Flow and the neural correlates. A) Contrast images with an FDR-corrected voxel *P* < 0.05 display significantly enhanced activation in *Cao-Shu* items compared with *Kai-Shu* items. Regions were found mostly distributed in the DAN core clusters by referring to Yeo’s DAN mask shown in the light red-shaded area. B) Change in functional interactions between 11 ROIs in *Cao-Shu* items compared with *Kai-Shu* items. Connections significant at uncorrected *P* < 0.01 were shown. C) Paired sample *t*-test shows higher flow in *Kai-Shu* items compared with *Cao-Shu* items. D) Flow negatively correlates with the activation in right FEF during calligraphic handwritings. E) Flow negatively correlates with the connectivity from VN to SMN during calligraphic handwritings. Each dot denotes 1 participant.

The following complementary whole-brain MVPA analyses further confirmed the wide involvement of the functional connectivity of regions in the VN, SMN, and DAN networks (Fig. 2E). Besides, the left orbital frontal cortex (OFC; MNI = [−18, 50, −6] and [−30, 50, −4]) and right caudate (MNI = [12, 20, 8]), which were key structures of the orbito-affective network (OAN; Haber and Knutson 2010; Ji et al. 2019) also showed significant differences between calligraphy writing and circle drawing tasks in their functional connectivity with the rest of the brain (thresholding at an uncorrected voxel of *P* < 0.001 and a cluster size of *k* > 10 voxels). Thus, in the later post hoc analyses, we particularly examined if the additional involvement of the OAN is related to pleasure during calligraphic handwritings.

### Flow experience during calligraphy

#### Higher flow in Kai-Shu handwriting

Considering that our participants had no formal calligraphy training experience, the difficulty of emulating *Kai-Shu* characters may have been more balanced with their skill level, and this could lead to a higher level of flow which will elicit more pleasure. As anticipated, performance data confirmed better skill-challenge balance in *Kai-Shu* items (*M* = 4.58, SD = 1.39) than that in *Cao-Shu* items (*M* = 3.84, SD = 1.54), *t* = 3.07, *P* = 0.007. Corresponding to the hypotheses, the paired samples *t*-test as shown in Fig. 3C demonstrated that the participants’ subjective ratings for flow experience among the *Kai-Shu* items (*M* = 5.08, SD = 0.18) were significantly higher than those among the *Cao-Shu* items (*M* = 4.63, SD = 0.19), *t* = 3.58, *P* = 0.002.

#### Less activation and connectivity in Kai-Shu handwriting

The contrast testing for significantly greater activation during *Cao-Shu* items compared with the *Kai-Shu* items based on the activation map of the calligraphy writing yielded a set of brain regions in the right angular and right precuneus cortex, left precentral gyrus, right superior frontal gyrus, and left middle occipital gyrus (*P* < 0.05, FDR-corrected), which are primarily located within the DAN, as confirmed by Yeo et al. (2011); as well as a further cluster in the supplementary motor cortex in *Cao-Shu* handwritings (*P* < 0.05, FDR corrected; Fig. 3A and Table S3). The gPPI analyses further shows higher connectivity between the VN and DAN during the *Cao-Shu* writing when compared with the *Kai-Shu* writing (from the right LVA to the left FEF: *t* (18) = 3.70, *P* = 0.002; from the OVA to the left FEF: *t* (18) = 3.39, *P* = 0.003; from the left LVA to the left FEF: *t* (18) = 3.14, *P* = 0.006; and from the left FEF to the OVA: *t* (18) = 3.02, *P* = 0.007, uncorrected; Fig. 3B).

When compared with the circle drawing task, the separate profiles of brain activation and connectivity in both *Kai-Shu* and *Cao-Shu* were very similar to the overall patterns (Fig. S1). To sum up, the *Kai-Shu* handwriting is characterized of a less effort/energy demanding brain activity pattern, i.e. smaller in activation amplitude and connectivity strength, whereas keeping the overall neural pattern unchanged.

#### Higher flow is associated with efficient (less) activity

To examine if the less activity represents an efficient pattern for higher flow experience, a series of correlation analyses between participants’ subjective flow scores during the calligraphy writing task and the corresponding effects at the neural level (i.e. the activation of 4 clusters within DAN shown in Table S2 and connectivity from VN to SMN at network level during the calligraphy writing task compared with the circle drawing task) was performed. The voxel coordinates of 4 activation clusters within DAN were extracted from the whole-brain group to contrast the calligraphy writing task > circle drawing task and used as the center coordinates for the spherical ROIs (6-mm radius; Table S2). We found that the flow scores for calligraphy writing were negatively correlated with the activation in the right FEF (MNI = [28, −6, 56], *r* = −0.47, *P* = 0.04; Fig. 3D) and the functional connectivity from the VN to SMN at the network level (*r* = −0.52, *P* = 0.02; Fig. 3E). It should be noted that the correlation coefficients between the activation in the regions within the DAN and the participants’ subjective flow ratings were uncorrected. Combing the results from both the comparison of brain activation and connectivity between 2 levels of skill-challenge balance of calligraphy, and the association between self-reported flow and these neural measures, we demonstrate that an efficiently working brain characterized as less demanding of brain activation and connectivity contributes to higher flow experience in calligraphic handwriting.

### Paths to pleasure

#### Contribution of reinforced OAN connectivity to pleasure

**Fig. 4:**
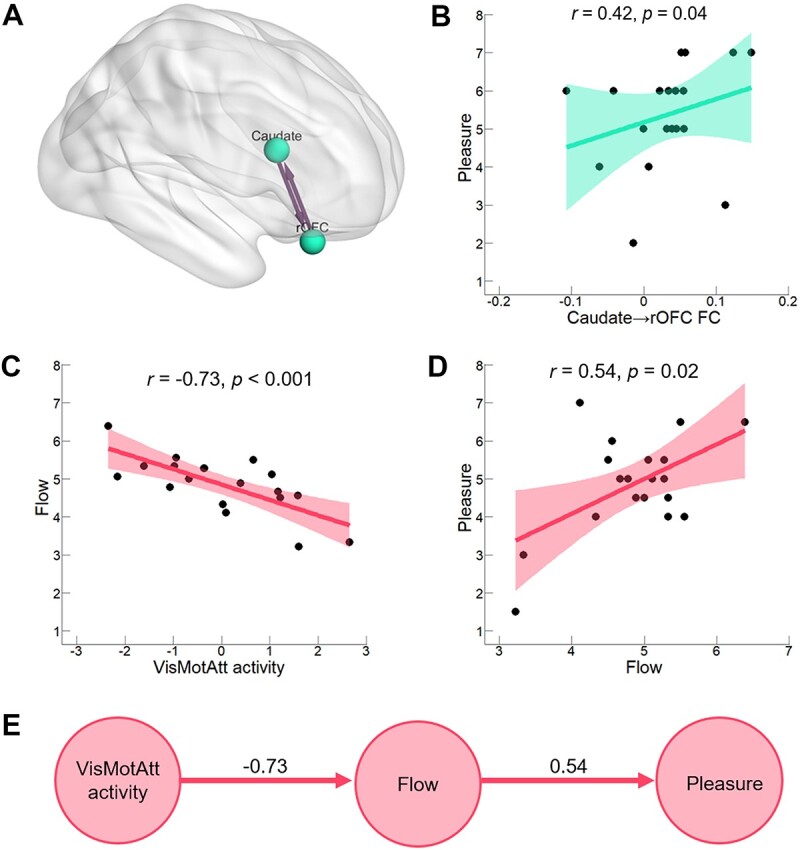
Neuropsychological paths to pleasure. A) Change in functional connectivity between right OFC and caudate in *Cao-Shu* items compared with *Kai-Shu* items. Connections significant at FDR-corrected *P* < 0.05. B) Pleasure positively correlates with connectivity from right caudate to OFC in *Kai-Shu* items. C) Correlation analysis shows a negative correlation between flow and VisMotAtt activity during calligraphic handwritings. D) Correlation analysis shows a positive correlation between pleasure and flow during calligraphic handwritings. E) The BIC-optimized GBN causal graph of brain activity, subjective flow, and pleasure. It is a compact representation of joint Gaussian distribution, and mathematically equivalent to a set of multivariate linear regression equations. The arc in the graph indicates the direction of causal relationship, and the number above represents the weight for the edge.

We first examined if the OAN as identified in the MVPA contributes to pleasure. Based on the behavioral data that there were higher levels of pleasure among the *Kai-Shu* items than that among the *Cao-Shu* items, *z* = 2.43, *P* = 0.01, we observed that the *Kai-Shu* items resulted in significantly increased functional connectivity between the right caudate and right OFC within the OAN (from the right OFC to the right caudate: *t* (18) = 2.74, *P* = 0.02; from the right caudate to the right OFC: *t* (18) = 2.56, *P* = 0.02; FDR corrected; Fig. 4A). Correlating the OAN connectivity with mean pleasure scores for 2 calligraphic styles did not yield significant relationship between higher functional connectivity and more pleasant experience for 2 calligraphy styles (from the right caudate to the right OFC, *r* = −0.02, *P* = 0.47; from the right OFC to the right caudate, *r* = −0.07, *P* = 0.39, one sided). However, when examining separately, a significant positive correlation was demonstrated between stronger connectivity from the right caudate to the right OFC and higher subjective pleasure ratings in *Kai-Shu* handwritings (*r* = 0.42, *P* = 0.04, one sided; Fig. 4B).

#### Visuomotor-attention network to mental flow that contributes to pleasure

Prior to estimating the GBN causal graph of flow, pleasure, and brain activity for calligraphic handwriting, we aggregated the flow-related activity measures, including the activation in the right FEF (MNI = [28, −6, 56]) and the functional connectivity from the VN to SMN (which both had negative correlations with flow) into 1 factor: the “VisMotAtt activity.” This represented a brain activity profile during calligraphy writing by standardizing the activation or connectivity scores into *z* scores, and then calculating the mean of the *z* scores. As shown in Fig. 4C and D, the VisMotAtt activity was negatively correlated with flow (*r* = −0.73, *P* < 0.001), which positively correlated with pleasure (*r* = 0.54, *P* = 0.02). Data of the 3 variables including subjective flow, pleasure, and VisMotAtt activity were entered into the GBN modeling procedure. Figure 4E shows the BIC-optimized GBN graph of 3 variable nodes, demonstrating a causal chain (or Markov chain) from VisMotAtt activity to flow then to pleasure for the calligraphic handwriting.

## Discussion

We investigated the neural mechanism that supports flow experience and pleasurable emotions using an imagined calligraphy writing task. Building upon the findings from neural studies of art, we confirmed that artistic engagement, and even imitation, recruited wide participation of different brain systems. Specifically, we demonstrated that calligraphy writing required the coactivation and cooperation of multimodal brain regions related to visual-motor integration, top-down attentional effort, and positive affect. Critically, we revealed that higher flow was linked to less demanding brain activity in terms of both activations, particularly in the brain regions within DAN, and functional connectivity, particularly between networks. The 2 lines of evidence intriguingly suggest a brain state that is busy and focused yet efficient in the flow state during calligraphy writing. Finally, we propose that pleasure from calligraphy handwriting arises from the efficient VisMotAtt circuit that enables the emergence of flow, and the orbito-caudate system that is responsible for feelings of affection. Taken together, these results provide new insight into the neural mechanism of flow and pleasure during artistic activities.

### Neural substrates for calligraphy

Relative to the circle drawing task, we observed increased activation in the VN and SMN, as well as stronger connectivity from the VN to the SMN. Participatory art, which emphasizes making art rather than observing art (e.g. writing calligraphy rather than appreciating it; Noice et al. 2014), is a highly complex skill-learning activity in which sensory and motor functions are closely linked (Bassett et al. 2015). Calligraphy writing requires fine-grained visual motor integration, and the soft-tipped writings place greater demand on gazing at the brush tip and the resultant handwriting, as well as on body control under visual guidance. The results show that the activated brain regions in the calligraphy writing overlap with the visual dorsal stream that connects the occipitoparietal cortex to the posterior half of the inferior parietal lobule. This stream is suggested as a neural substrate for supporting visually guided action (Kravitz et al. 2011; Mishkin and Ungerleider 1983), and has direct connections with the motor systems that control the head and eye movements, eye muscles, and the limbs (Baizer and Desimone 1993; Borra et al. 2014; Glickstein et al. 1985; Qian et al. 2016). Furthermore, its interconnections with the ventral stream also play an important role in regulating complex and flexible visuomotor skills (Milner 2017). Therefore, our exploratory findings suggest that the dorsal stream may be the crucial neural structure for visual-motor interactions in calligraphy training. Meanwhile, we found that the dorsal attention system that was mainly involved in the top-down orientation of attention (Fox et al. 2006) was also activated. The complexity of calligraphy writing places higher demand on attention, and screening-out distractions and staying focused is an indispensable state during calligraphy writing. Involvement of the DAN could serve to coordinate multistep behaviors and allocate processing resources to calligraphy-relevant stimuli, thus ensuring success in the calligraphy-writing process (Harris et al. 2017). Moreover, the further group-MVPA analyses confirmed the involvement of the orbito-affective circuit in *Kai-Shu* items, which indicates that an individual could emotionally engage in the calligraphy-writing process that better balance with their skills, despite the use of 12 valence-free Chinese ideographs as writing contents. Overall, the calligraphy writing process is supported by multiple brain networks, such as the visual and sensorimotor integration along a more dorsal stream, the top-down attentional control system, and the affective circuit.

### Neural activity pattern of flow

We observed that *Kai-Shu* induced higher flow and brought about less brain activation and network connectivity, which suggested that there was a relationship between higher flow and downregulated brain activity. However, according to traditional models of attention that more difficult tasks may require greater demands for mental energy (McGuire and Botvinick 2010), the downregulated brain activity during the *Kai-Shu* items could be a result of the lower difficulty. Then a series of correlation analyses was conducted to further examine the relationship between brain activity and flow experience. Overall, the VisMotAtt activity was negatively correlated with flow experience. Specifically, functional connectivity from the VN to the SMN and activation in the DAN were both negatively correlated with flow level in the calligraphy writing conditions. These findings indicate that a less effort/energy demanding brain activity pattern may be optimal for experiencing flow during calligraphy writing. Psychophysiological studies also suggest that excessive physiological arousal could hinder flow state (Peifer et al. 2014; Tozman et al. 2015). Using graph theoretical analyses, Huskey et al. (2018b) also found highest average network degree in the frontoparietal control network and lowest global efficiency, which indicates low metabolic cost in flow condition. Tasks in prior studies were set in 3 conditions including flow (difficulty ≈ ability), overload (with very high task difficulty), and bored conditions (with very low task demands; Ulrich et al. 2014, 2015; Huskey et al. 2018a), and no linear relationship was investigated or observed between flow scores and key brain regions. Neural activity pattern of flow was investigated through comparison among those different conditions instead of linking brain activity to flow experience. In this study, calligraphy art is regarded as a flow-inducing activity based on its characteristics without specific dividing lines among balanced, overload, and bored conditions and scores of flow are used to index the experiential quality of artistic activity (de Manzano et al. 2010).

The previous studies have revealed the role of the sensory-motor network activity in terms of flow contribution (Klasen et al. 2012), and the current results emphasize the importance of efficient sensory-motor connectivity. Moreover, the relative increase of neural activation in the DAN compared with circle drawing may support the idea that flow is not completely automatic but demands attentional effort (Harris et al. 2017). During complex calligraphy writing, top-down attentional effort is required, which can be highly demanding. During flow, however, attentional control is goal-directed and more dependent on the absence of stimulus-driven disruption and monitoring processes (Harris et al. 2017); therefore, being focused but effortless may constitute an intriguing feature of flow during calligraphy writing. Similar results have also been observed in sports (Li and Smith 2021) as well as in other artistic activities, such as chess (Grabner et al. 2006), and have revealed that experts show lower activation in task-related brain regions than less skilled individuals.

Furthermore, consistent with the previous findings (Ulrich et al. 2014; Huskey et al. 2018a), the involvement of the putamen, a key component of dorsal striatum, was observed during the calligraphy writing. This structure is implicated in the coding outcome probability to regulate rewarding experiences in tasks that balance one’s skills with the task challenges, which could support the autotelic nature of flow experience (Satterthwaite et al. 2007; Ulrich et al. 2015, 2016; Melnikoff et al. 2022). Moreover, the dorsal striatum has been associated with automatized implicit processing (Gold and Ciorciari 2020), which may suggest that the effortless attention and lack of conscious thought during calligraphy writing are consequences of the transition from explicit executive functions to a faster implicit system.

### Neuropsychological mechanism of pleasure

Calligraphy writing has been found to improve mood (Kao 2006; Xu et al. 2013), and the current study’s findings provide insight into the neuropsychological underpinnings of pleasurable emotions in calligraphy. Flow state induced during calligraphy writing appears to promote pleasurable emotions, which are characterized by enhanced connectivity within the orbito-affective circuit at the neural level. In addition to the affective-related subcortical structure, obtaining pleasure in calligraphy activity also involves orbitofrontal cortex, which could implement attractor networks useful in maintaining reward and in decision-making (Rolls et al. 2020). A similar pattern that frontal cortex increased connectivity with the nucleus accumbens (NAcc) has also been observed during music processing (Salimpoor et al. 2013). Differing from the immediate and short-term rewards brought about by low-level perceptual stimuli, higher-order pleasure engages widespread neural systems to support the flow state alongside the reward circuitry (Christensen 2017). Rewarding activities that are mainly mediated by the amygdala and striatum, such as playing games, gambling, hyper-palatable foods, sex, and pornography, lead to cravings that could strip individuals of their willpower to make good choices (Bechara 2005; Goldstein and Volkow 2011; Wilcox et al. 2014; Christensen 2017). Whereas, pleasurable experiences during artistic activities also engage top-down regulatory networks for rational decision-making (Salimpoor et al. 2013; Christensen 2017). Different from other animals, human beings can obtain pleasure from complicated and highly abstract activities that are dependent on cultures thanks to more evolved brain regions, particularly the prefrontal cortex (Cela-Conde et al. 2004). It is proposed that pleasure in these activities arises from cooperation between highly evolved cerebral cortex that could relate abstract stimulus to earlier experience and generate expectations about upcoming events and subcortical systems that assess the outcome of these predictions (Zatorre and Salimpoor 2013). The cortical loops are engaged when activities trigger memory and frontal experiential systems, and it may form the basis of pleasure wanting derived from anticipation and a sense of meaningfulness (Christensen 2017; Stark et al. 2018). Christensen (2017) proposes to refer to pleasurable state of flow as the marker of engagement of both cortical and subcortical rewarding systems. Huskey et al. (2018a) have found stronger functional connectivity between NAcc and the dorsal lateral prefrontal cortex in flow state. This kind of “higher-order” pleasure during flow would not induce states of craving without fulfillment or cause addiction; instead, it could contribute to maintain long-term prosperity (Stark et al. 2018). Furthermore, developing the BIC-optimized GBN causal graph of brain activity, subjective flow, and pleasure, we show that an efficiently working brain is optimal to experience flow, which then contribute to pleasurable emotions. These findings highlight the important role of flow in experienced pleasure during artistic activities and corresponding neural effects, which provide further support and extension for synchronization theory of flow.

### Prospect of calligraphy on mental health

Calligraphy writing could induce flow as mediated by the efficient integration of visuo-motor systems with effortless concentration to elicit a pleasurable experience. The various ways of writing in calligraphy (e.g. tracing, copying, or free-form writing) and diverse calligraphic forms at different difficulty levels can be dynamically balanced with one’s skill level, thus contributing to continuous flow that imbues life with pleasure and leads to long-term prosperity (Stark et al. 2018). There is also evidence that recited poetry can induce peak emotional responses including feelings of chills and the involvement of the NAcc (Wassiliwizky et al. 2017). Our results may suggest that calligraphy has a strong positive effect on assisting individuals to experience higher-order pleasure that is full of meaningfulness. The diversity of calligraphy makes it possible for an individual to reach a dynamic skill-task challenge balance during the different stages of calligraphy learning. Moreover, experienced pleasure may contribute to the enhanced motivation to practice calligraphy. More positive emotions mean more practice, thus starting the positive feedback loop. Overall, our findings shed light on the neurological substrate of flow and pleasure in calligraphy writing and highlight the function of calligraphy in mental health protection. Going forward, we call for the future research to utilize localized artistic activities, such as the Chinese calligraphic handwriting as intervention tools to promote the cognitive and emotional health of specific populations through longitudinal studies.

## Limitations and future study

First, a limitation of the current study was that the participants had no formal calligraphy training, and their lack of experience may have limited the vividness of their imagination. Regardless, compared with real calligraphy writing, the imagined paradigm used herein could provide better balance between subjective challenges and skills for calligraphy novices. Moreover, this may increase the generalizability of the results in a large sample and may provide powerful evidence to better advocate for the practice of Chinese calligraphy. Second, our exploratory research has showed several interesting findings, but the small sample size is likely to yield low statistical power and cause possibility to overlook potential relationships. It is necessary in the future to validate and extend present findings in larger and more diverse samples. Third, circle drawing task in the experiment is set up to control the activation elicited by general visual and motor processing. However, on the other hand, it may potentially induce some psychological effects such as stress or relaxation which have not been considered in the present study. Lines or patterns resembling Chinese characters could be designed in future studies to better explore the uniqueness of calligraphy in neural activation and connectivity pattern. Fourth, the computational model from the data-driven learning suggests possible neuropsychological paths from brain activity to flow state that further produces pleasure, but this model does not depict a complete relationship among them. Strict randomized controlled trial and a longitudinal design could be used in future studies to examine causal relationships between brain function and structure with these psychological variables. The major challenge of investigating the neural mechanisms of calligraphy concerns the difficulty of performing tasks in the fMRI environment. Based on the embodied cognition theory, body posture and movement during calligraphy writing may be key factors that mediate the effects of calligraphy on mental health (Koch 2014; Skulmowski and Rey 2018). Calligraphy includes not only the movement of fingers, wrists, and arms, but also the movement of the whole body for large pieces of writing; all of which cannot be fully studied in an fMRI environment. In the future, it will be necessary to use wearable and mobile devices to further explore the neurophysiological activities during real calligraphy writing to better understand the flow experience. The current study should be treated as exploratory, and it is hoped that the findings can be replicated in further studies to support the robustness of the results. To explore training-related changes, it would be valuable to compare the differences in the neurological and psychosocial variables during the calligraphy writing process among different stages of calligraphy learning.

## Conclusion

This study explores how the neural network interactions support mental flow that stimulates pleasurable emotions during artistic activities. Using an imitation task of calligraphic handwriting, we demonstrate an efficiently processing brain characterized as a less effort/energy demanding brain activity pattern contributes to higher flow experience. Furthermore, we demonstrate that pleasure in the calligraphy task arises from the efficient cortical activity in the emergence of flow, and the enhanced orbito-caudate affective network. These findings provide new insight into the neuropsychological mechanism of mental flow in artistic activities, and can spur new applications of artistic activities, such as calligraphic handwriting, in clinical therapies to boost well-being.

## CRediT authors statement

Yue Wang (Conceptualization, Data curation, Formal analysis, Investigation, Methodology, Resources, Software, Validation, Visualization, Writing—original draft, Writing—review & editing), Buxin Han (Conceptualization, Project administration, Supervision), Min Li (Resources, Supervision), Juan Li (Supervision), and Rui Li (Conceptualization, Data curation, Formal analysis, Funding acquisition, Investigation, Methodology, Project administration, Resources, Software, Supervision, Validation, Visualization, Writing—original draft, Writing—review & editing)

## Funding

The Scientific Foundation of Institute of Psychology, Chinese Academy of Sciences (E0CX121008, E2CX3715CX); the National Natural Science Foundation of China (61673374). The funders had no role in study design, data collection and analysis, decision to publish or preparation of the manuscript.

*Conflict of interest statement*: None declared.

## Data availability

All data generated or analyzed during this study are available from the corresponding author on reasonable request.

## Supplementary Material

Supplementary_Material_bhad047
