# Supplementary material for: An efficiently working brain characterizes higher mental flow that elicits pleasure in Chinese calligraphic handwriting
Source: Cereb Cortex. 2023 Mar 9;33(12):7395–408. doi: 10.1093/cercor/bhad047 (PMC10267646; doi:10.1093/cercor/bhad047)
Supplement: Supplementary_Material_bhad047 [file supplementary_material_bhad047.docx]

**Supplemental Material**

**Table S1** Character lengths, frequencies ^a^, and durations of video clips

| Style | Character | Character length | Character frequency/million | Duration(s) |
| --- | --- | --- | --- | --- |
| *Kai-Shu* | 者 | 8 | 901.88 | 26 |
|  | 物 | 8 | 642.15 | 24 |
|  | 照 | 13 | 517.77 | 32 |
|  | 典 | 8 | 69.94 | 26 |
|  | 泉 | 9 | 19.58 | 26 |
|  | 郡 | 9 | 19.17 | 26 |
|  | 冠 | 9 | 63.98 | 30 |
|  | 坤 | 8 | 4.76 | 22 |
| *Cao-Shu* | 理 | 11 | 877.41 | 24 |
|  | 将 | 9 | 650.28 | 34 |
|  | 周 | 8 | 450.29 | 28 |
|  | 委 | 8 | 110.65 | 22 |
|  | 势 | 8 | 97.07 | 26 |
|  | 静 | 14 | 244.38 | 26 |
|  | 雪 | 11 | 79.65 | 22 |
|  | 清 | 11 | 462.41 | 24 |

a. Statistical data from the database of word and character frequencies based on a corpus of film and television subtitles (Cai and Byrsbaert, 2010)

**Table S2** Regions significantly activated in calligraphic handwritings

|  | Coordinate (MNI) | | |  |  |
| --- | --- | --- | --- | --- | --- |
| Region | x | y | z | *t*-value | *K*_E_ |
| **Dorsal attention network** |  |  |  |  |  |
| left intraparietal sulcus | -24 | -52 | 46 | 12.16 | 2358 |
| right intraparietal sulcus | 30 | -58 | 50 | 13.81 | 2758 |
| left frontal eye field | -24 | -6 | 54 | 13.69 | 1235 |
| right frontal eye field | 28 | -6 | 56 | 11.97 | 1339 |
| **Visual network** |  |  |  |  |  |
| left occipitotemporal cortex | -42 | -58 | -18 | 13.21 | 1183 |
| right occipitotemporal cortex | 46 | -54 | -18 | 8.40 | 811 |
| **Sensorimotor network** |  |  |  |  |  |
| left precentral gyrus | -18 | -10 | 64 | 9.13 | 1130 |
| right precentral gyrus | 12 | -6 | 66 | 9.39 | 1130 |
| left postcentral gyrus | -32 | -40 | 52 | 8.52 | 734 |
| right postcentral gyrus | 42 | -30 | 46 | 5.54 | 408 |
| **left putamen/** **thalamus** | -20 | 0 | 12 | 6.14 | 730 |
| **right putamen/** **thalamus** | 16 | -22 | 8 | 5.70 | 523 |

**Table S3** Regions showing significant activation difference between *Cao-Shu* and *Kai-Shu* handwritings

|  | Coordinate (MNI) | | |  |  |
| --- | --- | --- | --- | --- | --- |
| Region | x | y | z | *t*-value | *K*_E_ |
| **DAN rIPS cluster** |  |  |  |  |  |
| right angular | 30 | -54 | 32 | 7.27 | 138 |
| right precuneus cortex | 12 | -60 | 48 | 4.50 | 20 |
| right precuneus cortex | 10 | -70 | 56 | 4.45 | 20 |
| **DAN lIPS cluster** |  |  |  |  |  |
| left middle occipital gyrus | -26 | -64 | 36 | 6.75 | 90 |
| **DAN rFEF cluster** |  |  |  |  |  |
| right superior frontal gyrus | 24 | 2 | 66 | 4.80 | 37 |
| **DAN lFEF cluster** |  |  |  |  |  |
| left precentral gyrus | -38 | 2 | 32 | 5.07 | 41 |
| **Supplementary motor cortex** | -6 | 8 | 54 | 5.29 | 55 |

**Figure. S1** Functional activation patterns in the *Kai-Shu* and *Cao-Shu* items superimposed with overall patterns.
